# Novice Experts: Exploring Fellows’ Perspectives on the Transition from Residency to Fellowship

**DOI:** 10.5334/pme.1654

**Published:** 2025-02-14

**Authors:** Laura E. Chiel, Michael Fishman, Erik Driessen, Ariel S. Winn

**Affiliations:** 1 Division of Pulmonary Medicine, Department of Pediatrics, Boston Children’s Hospital and Harvard Medical School, 300 Longwood Ave., Boston Children’s Hospital, Boston, MA 02115, US; 2 School of Health Professions Education (SHE), Maastricht University, Maastricht, NL; 3 Division of Emergency Medicine, Department of Pediatrics, Boston Children’s Hospital and Harvard Medical School, 300 Longwood Ave., Boston Children’s Hospital, Boston, MA 02115, US; 4 School of Health Professions Education (SHE), Faculty of Health, Medicine and Life Sciences of Maastricht University, Department of Educational Research and Development, P.O. Box 616, 6200 MD Maastricht, NL; 5 Division of General Pediatrics, Department of Pediatrics, Boston Children’s Hospital and Harvard Medical School, 300 Longwood Ave., Boston Children’s Hospital, Boston, MA 02115, US

## Abstract

**Introduction:**

Advanced training experiences are required in certain countries for subspecialization. In the United States, a decline in Milestones and in levels of supervision for Entrustable Professional Activities for incoming subspecialty fellows has been described and attributed to changes in context that fellows experience. We aimed to explore this transition to advanced training, and specifically to describe which contextual factors are salient to fellows at the residency to fellowship transition and the supports available for a smooth transition to fellowship.

**Methods:**

Using contextual competence as a sensitizing concept, ten semi-structured interviews with first- and second-year pediatric subspecialty fellows from three subspecialties were performed at a large academic medical center in 2023, using thematic analysis informed by elements of constructivist grounded theory.

**Results:**

Contextual factors that impacted the transition included changes in systems, necessary knowledge, and roles and responsibilities. At times, participants describe a tension between feeling like novices while simultaneously feeling like they should have more expertise than they had. Supports in navigating this tension, and in navigating the transition more generally, included formal orientations, fellow behaviors and perspective, and input from others.

**Conclusions:**

The transition to advanced training is characterized, at times, by experiencing tension between feeling like a novice while feeling like one should have expertise, with fellows’ own behaviors and the support of those around them being essential to fellows’ smooth transition. While fellowship programs offer orientations, systems-level solutions for supporting fellows’ navigation of the transition are underexplored.

## Introduction

Clinical fellowships, or periods of advanced training after completion of initial training and attaining the level of independent practitioner, are required for subspecialization in certain medical education systems globally. For example, in the United States (US), after a three-year training program in pediatrics, after which the trainee can practice independently as a pediatrician, an additional three-year fellowship is required for subspecialization, for example in pediatric pulmonology or pediatric gastroenterology. Similarly, after pediatrics training in Germany, trainees may go on to pursue additional titles in specialty fields. Although trainees pursuing subspecialty training are advanced in their learning and their experience of being a trainee, there is literature that suggests that this transition to advanced training is not seamless.

An analysis of the United States’s Accreditation Council for Graduate Medical Education (ACGME) Milestones common to pediatrics residency and fellowship revealed significantly lower scores for early fellowship compared to late residency [1], and a study on common Entrustable Professional Activities (EPAs) between residency and fellowship found that early fellows needed more supervision than graduating residents and, at times, more than early residents [2]. In explaining their results, both Reed et al [1], and Schwartz et al [2], implicate ‘context’ as the reason Milestones and EPAs decline. Reed et al. found that while the pattern of ACGME Milestone decline persisted, trainees who stayed at their residency institution for fellowship had less decline, suggesting the importance of context in transition [1]. Schwartz et al. noted that in the fellowship setting, ‘the scope and stakes of practice advance’ and that supervisors ‘may prefer to rely on more proximate and contextually bound assessments before making entrustment decisions’ [2]. Thus, changes in context may be a key driver in trainee performance in the transition to advanced training.

For purposes of this study, we use Bates and Ellaway’s definition of context, which was derived based on a thorough review of other definitions of context in the literature, as follows: ‘*Context* is a dynamic and ever-changing system that emerges from underlying patterns of patients, locations, practice, education and society, and from the unpredictable interactions between these patterns’ [3]. That context is an important mediator of transition experiences is well described in other transition points in medical training [4,5,6,7,8,9,10]. In a qualitative study examining preparation of new consultants for their roles, participants shared a number of contextual factors impacting their transition experience, including the need to navigate new personal situations, organizational structures, and cultures, potentially with varying degrees of support [9]. New consultants’ familiarity with the department and hospital has been linked with comfort transitioning into supervisory roles [10].

Context and competence are known to be inextricably tied, with competence described not as an ‘intrinsic characteristic of an individual’ but as an ‘enactment of optimal (or near optimal) performance expressed in and for a particular context’ [11,12]. Teunissen and colleagues have begun to define how learners regain prior competence when transitioning between residency rotations, via their concept of ‘contextual competence’ [13]. Specifically, they describe Bates’ hierarchy of contextual competence, from establishing basic physiological and practical needs, to developing legitimacy and belonging, to ultimately developing competence and autonomy [13]. The development of contextual competence has been described within residency programs [13]; there is a gap in our knowledge as to which contextual factors are most salient at the transition to advanced training programs and how trainees navigate these factors to smoothly transition.

Using contextual competence as a sensitizing concept, in this study we explore this transition by more closely examining the transition from US-based residency to fellowship programs, seeking to explore from the perspective of fellows: (1) What are the salient contextual factors at the transition from residency to pediatric subspecialty fellowship? and (2) What are the supports to a smooth transition to pediatric subspecialty fellowship?

## Methods

### Study Design

We approach this study from a constructivist paradigm with the vantage point that knowledge is socially constructed [14]. We performed a thematic analysis, informed by elements of constructivist grounded theory methodology, to delve into the not well-understood social phenomenon of transition from residency into fellowship [15].

### Participants

We recruited participants from three fellowship programs at Boston Children’s Hospital, a large academic medical center. These fellowship programs were selected as they represent distinct contexts. The Pediatric Emergency Medicine (PEM) Fellowship is an 18-fellow program where fellows predominantly practice in the pediatric emergency department, with briefer rotations in an adult emergency department, the operating room, and in the intensive care unit. The Cardiology Fellowship is a 24-fellow program where fellows practice inpatient and outpatient care, perform consults in the emergency department, participate in catheterization-based procedures, and take call from home. The Critical Care Medicine (CCM) fellowship is an 18-fellow program where fellows predominantly practice in the intensive care unit.

We purposively sampled fellows to ensure a mix of those who completed residency at the study institution and those who did not. We included first-year fellows, actively in the process of navigating transition into fellowship, and second-year fellows who are more advanced in the transition process. We selected participants who had completed a chief resident year during which they served as an attending physician and a participant who completed a residency that involved caring for both adults and children. These individuals were selected to provide a sample of those who would be entering the selected fellowships with a more complete range of previous experiences. L.C. sent a recruitment email to the participating fellowship program directors with the request to forward to first and second-year fellows in their programs. A gift card was provided to study participants as a token of appreciation.

### Data Collection

In 2023, we interviewed ten participants, with at least two participants from each fellowship program. Six participants identify as women. Six participants completed residency at the same institution where they are completing fellowship. Three participants completed a chief resident year and one participant completed a residency in which they cared both for adults and children. Five participants were in their first year of fellowship and had not completed any additional training.

Data was collected through semi-structured interviews via Zoom. L.C., who has no role in assessing fellows in the selected fellowship programs, served as the interviewer. The interviews were based on an interview guide which was developed by the study team about contextual competence and factors affecting the transition. The guide was piloted with two second-year fellows from a non-participating fellowship program and updated for clarity (Supplementary Material) and further updated iteratively throughout the study. Interview duration was maximally one hour (35–51 minutes, excluding study introduction, verbal consent, and closing). Interviews stopped when LC noticed from participants’ responses that thematic sufficiency was realized.

### Data Analysis

ATLAS.ti (Lumivero; Berlin, Germany) was used to help facilitate analysis. L.C. coded the first two interview transcripts and shared the transcripts and an initial codebook with the team for feedback and additional interpretations, after which she continued to iteratively code the remainder of the transcripts, developing concepts through constant comparison. Near completion of the initial coding, she shared two transcripts with M.F. and two different transcripts with A.S.W., as well as early concepts from the analysis, so as to inform research team discussions. The research team met frequently to review connections between codes, to establish concepts, and diagrams of how concepts related, facilitating further dimensionality to the interpretation. During iterative analysis, L.C., in discussion with the study team, determined how to enrich the findings that were developing through further sampling [15]. The authors reflected on their orientations to the topic and shared memos about how their own backgrounds influenced their interpretations, adding depth to the analysis [16]. L.C. noted her tendency to “side with” the fellow experience having been a recent fellow while aligning with programs as a current fellowship program leader. M.F. brought his stance as a current fellow to the analysis. A.S.W used her experience supervising fellows to inform the analysis. E.D. does not work clinically with fellows and thus was able to maintain an “outsider’s” view. The study received Institutional Review Board exemption at Boston Children’s Hospital.

## Results

We describe the key contextual factors in the transition from residency to fellowship, including systems, necessary knowledge, and new roles and responsibilities. We describe a tension that participants faced, at times, between feeling like novices and simultaneously feeling like they should have more expertise. We then describe supports that help ease this tension, and the transition more broadly, either through helping participants gain new skills that lead them to feel more expert, by changing their perception of the degree of expertise that a fellow should have, or a combination of the two. These supports fit into three categories: (1) orientations; (2) fellows’ own actions; and (3) input from others.

### Key contextual factors in the transition from residency to fellowship

#### Systems Can Serve as Barriers

Participants describe systems-related barriers to working effectively, with one noting that ‘I think finding where things are is really hard — I really wish there was a map’ (P5) and another describing getting ‘stuck’ in the electronic medical record, sharing ‘and you just feel like you’re treading through molasses and it can be very frustrating’ (P1). Participants rotate across a number of services, each with its own specific systems-related complexity, with a participant explaining ‘and it honestly almost feels like residency again, where you’re starting from scratch every month so that that was a little challenging, and having to go through the motions of learning all the logistics for that rotation every month’ (P5). In addition to acknowledging systems challenges within the hospital, participants also navigated systems challenges in acclimating to a new city. One participant explained, ‘That was a nightmare, but like parking that car and like getting it ready, that all took, like most of my free time that first month, like just figuring out parking and registering my car’ (P5).

#### New Necessary Knowledge and Steepness of the Learning Curve

At the same time as familiarizing themselves with new systems, participants describe being put on a ‘learning curve’ that is ‘very steep’ (P4), with the medical knowledge needed to competently care for patients becoming increasingly subspecialized with the transition to a new context. A participant explained that it was ‘tough’ to go from ‘feeling pretty competent, as like a senior pediatric resident’ to ‘like not knowing some of the most basic parts of the field’ (P1).

Another participant elaborated: “I need to have more knowledge in my specific specialty [cardiology]…if I tell an attending ‘Oh yeah, I hear a systolic murmur here,’ and they’re like, ‘no, you gotta tell me more. Is it high pitched? Is it musical, vibratory? Where? Does it radiate anywhere else? Does it sound like?… So I think pushing myself…what else do I need to know as a fellow… as a cardiologist” (P2).

#### Managing Multiple Conflicting Roles While Assuming New Responsibilities

Participants describe acclimating to a new role and depending on the fellowship, multiple roles, including as learners, team leaders, educators, proceduralists, and consultants. A participant described their transition into becoming a team leader as ‘stepping more into the role of globally looking at a patient rather than just focusing on like a small, tiny piece of the puzzle and changing from presenting the data to really being responsible for interpreting all of that data and understanding the global context’ (P9), while another described stepping into the role of educator as ‘being almost like a translator from an educational perspective’ between a ‘very seasoned attending’ and a new learner who is trying to understand the ‘alphabet soup’ of acronyms in the specialty, when ‘obviously the letters are just starting to form meaning for me as an early fellow’ (P4).

In tandem with owning new roles, participants describe feeling new responsibility for patients. A participant shared that their ‘feeling of responsibility for what happens to my patients has gone up even farther than it was in residency, and I think in terms of the question of like in 2 years, I’m gonna be the one whose name is the final name on this paper, on this discharge’ (P6). While in many cases participants felt greater responsibility as compared to residency, they shared that responsibility could vary from rotation to rotation, with a participant describing how this ‘can be so discombobulating,’ noting, “It’s like one minute you feel this huge amount of responsibility and … Suddenly you’re like, ‘Wait. I don’t get to make that decision, and you know I don’t have that responsibility,’ and I think that disconnect can feel very jarring as a fellow” (P4).

### Tension between feeling like a novice while simultaneously feeling like one should have expertise

Participants shared that, at times, they experienced tension between feeling like novices while feeling like they should have expertise or, more specifically, a tension between how they functioned in the system, their level of knowledge, or what they could do with how they perceived they should be able to function, what they should know, or what they should be able to do. This tension could be fueled by others or by the participants themselves. A participant highlighted fellows’ tendency to exacerbate this tension, explaining, “I think we all have those moments where you feel like a complete idiot where I think we’re all very much inclined to focus on the things we don’t know and ignore the things we do know” (P4). Other participants highlighted others’ contributions to this tension, noting, “Even, like, attendings in the ED would come to me directly and ask me a question, whereas, you know they know that I don’t really know anything yet about Cardiology. But they’d be like ‘hey, what do you think of the EKG’, I’m like, you probably have seen more EKGs than I have in my lifetime and it was very daunting and definitely felt like I was starting back as like a first year intern again” (P2). Another participant elaborated, ‘and everyone looks at you, and they assume you know what you’re talking about, and they assume you know what you’re doing’ while internally wondering, ‘do I actually know what I’m talking about?’ (P9).

### Orientations, fellow actions, and input from others support fellows in navigating the tension and in the transition more broadly

#### Orientations are “the Most Helpful Thing”

Participants described orientations as the most important systems-based intervention, allowing initial exposure to much of the content and systems knowledge they would be expected to attain in the months to follow. One participant described orientation as ‘the most helpful thing’ in that ‘it was like a first-time pass of, like all the content that we’re like going to be re-exposed to repeatedly throughout the next couple of years’ with the caveat that, ‘you can’t remember everything that [we] were taught in orientation, but at least we were exposed to the different fields’ (P1). Another explained ‘I felt like it set the precedent from the beginning that the program was invested in our education and not just our role as providing service’ (P4). Thus, orientations helped participants gain increased systems and clinical knowledge and sense of their roles, while also emphasizing fellows’ role as learners.

#### Fellows Can Take Action to Ease the Transition

Participants shared specific behaviors they take to smooth their transition as well as barriers to doing so. Some participants described, for example, the importance of preparation and self-study, with a participant describing ‘I push myself to learn as much as I can and read and study as much as I can’ (P8). Participants also described personal life factors – including getting acclimated to new settings, developing new routines, and balancing family responsibilities as helping or hindering their transition to fellowship.

While participants were motivated to further their development, they also described the importance of contextualizing for themselves the challenges of their conflicting roles and the expectations for increased knowledge and, in the process, reminding themselves they need not yet be experts. One participant framed how they navigated their conflicting roles: ‘Okay, yes, as a fellow, I should like walk in and have a framework, and like, know the one liner and be able to help a little bit. But ultimately, like again, the goal is not – don’t look dumb — the goal is, keep patient safe’ (P4), although shared that having this mindset was easier said than done. Another participant explained that comparing themselves to others could help them right-size what they should know, noting that it can be valuable to see that other fellows have knowledge gaps too, explaining ‘we’re at conferences with the different year fellows and it’s really nice knowing how the third years are thinking about things… and it’s okay to still have those kinds of questions as a third year,’ elaborating ‘at some point we’ll get it all, and we just have to trust the process – I guess, lifelong learning’ (P2).

#### Team Members Help Fellows Navigate the Transition

Attendings (consultants), near peers, and interdisciplinary team members within the field could share resources, teach, and otherwise create learning opportunities, facilitating a fellow’s development of new skills. A participant noted, ‘and I remember as soon as the patient was roomed and they knew there was a pneumothorax [the attending] immediately called me, and I just was really struck by the fact that whenever there’s something that was interesting, or complicated, or rare…it’s kind of like they run their ABCs and do DEF [ABCDE is an acronym used in critical situations], F for fellow, and always just seem to call you’ (P7).

Participants describe working with team members in the same specialty, including attendings, near peers, and members of the interprofessional team, as helping them clarify what they were and were not expected to know as new fellows, which was an important way to benchmark what level of competence they should have or strive for, and was a different type of support as compared to creating learning opportunities. Participants commented that those within the subspeciality often conveyed that ‘you’re not expected to know anything’ (P7), with another elaborating that the expectation from ‘program leadership and division leadership…is that we are learners first’ (P8). Others described the value of feedback and encouragement, with one participant describing how a sonographer allowed her to independently perform a scan and ‘afterwards she made a point to say that I did a really good job… and that positive reinforcement definitely made me feel good, like I can do this, like I can actually learn how to do this’ (P1). While team members often helped fellows navigate the tension they experienced, they could also exacerbate it. Participants noted that, at times, they did not have the same degree of autonomy that they had during residency, for example due to supervisors’ lack of familiarity with them and need for more time worked together to gain entrustment.

## Discussion

In this study, we described that pediatric subspecialty fellows navigate changes in key contextual factors – systems, necessary knowledge, and role and responsibility. Participants shared the tension they faced between being novices in their subspecialty while feeling, in certain circumstances, that they should have increased expertise. We found that orientations, fellow behavior and perspectives, and input from others help support fellows’ transition within a new context, either by furthering fellows’ expertise or by helping fellows understand that they need not be experts yet. Our findings overlap with prior work exploring the transition from training to independent practice in which one’s preparedness and personal characteristics, as well as familiarity with a hospital system and care for patients similar to those cared for previously were found to be key features of the transition [10]. The simultaneous positions adopted by the fellow of being both novice and expert, however, appears to be unique given the demands of new knowledge and multiple roles, in addition to new systems and more responsibility.

While in Bates’ hierarchy of contextual competence, ‘competence’ is generally something to be attained after navigating physiological needs and practical needs and establishing legitimacy and belonging, we found that advanced trainees were simultaneously navigating multiple contextual factors and seeking to hone various facets of competence all at once, whether gaining expertise in specialty-specific knowledge or seeking to act in a subspecialist role. This conceptualization of competence as multifaceted aligns with ten Cate and colleagues’ description of three layers of competence which are developed in stages throughout one’s career, including “canonical knowledge and skill” or “that, which every professional should possess”, “context-dependent knowledge, skill, and attitude,” and “personalised competence,” involving “personal skills, interests, habits and convictions, integrated with one’s personality” [17]. In our study, participants described the need to learn the knowledge and procedures of the subspecialty (canonical knowledge and skill) and how to do so within a particular system (context-dependent knowledge, skill, and attitude). In ten Cate and colleagues’ work, these layers of competence are often honed sequentially, with contextual and personalised competence layers becoming more salient in postgraduate education [17]. We found that there was a “reset” upon transition to advanced training and that canonical competence was again critical, suggesting that the gains made in prior training could only take incoming trainees so far, likely contributing to the experience of feeling inexpert.

Aside from orientations, there were few systems-level interventions noted to facilitate the transition, especially those that are designed for the fellow themselves or those in their close proximity, a group noted to have a significant impact on fellows’ transition. To bolster support provided by others, formal faculty development and education programs could be developed to train attending physicians, peers, and other health professionals how best to support incoming fellows both within and outside their subspeciality. These programs could target strategies to assist with the steep knowledge curve fellows face and to ensure a shared understanding of fellow expectations. Rather than leaving fellows to devise their own strategies to support their own transition, learner education handovers or formal coaching programs could be considered to help learners articulate their goals and develop strategies to meet them with the support of faculty. Learner educational handovers, or “the sharing of information about the learner across educational phases and supervisors” [18], are beginning to be implemented at the transition from medical school to residency in recognition of the disjointed nature of the transition [19,20,21,22], but have yet to be explored at this transition. Coaching programs are increasingly popular in medical school and residency programs, allowing learners to define goals and work to meet them with the support of a trusted faculty member [23,24,25], but are not widely used in pediatric subspecialty fellowships. Systems-level interventions such as refresher orientations, faculty development, coaching programs and learner handovers should be further explored.

### Limitations

We conducted our study in three fellowship programs at one US-based hospital to support study feasibility. Although we sought to balance inclusion of subspecialties with a variety of practice settings, and although there is evidence that even within the same hospital there are a number of contexts [1,2], we recognize that the sampling strategy poses a limitation in transferability to programs in other countries, at other institutions, and other subspecialty fellowships, particularly smaller, primarily outpatient-based specialties. We acknowledge that trainees in larger programs may have more peer support than those in small programs which may influence the transition experience. Programs and trainees will need to weigh the transferability to their own setting. Further, there is evidence that trainees have differing preferences when it comes to support at transitions [26], and larger studies may enrich our understanding. Additionally, larger studies may better clarify the impact of staying in the same institution and/or completing a chief resident year, where one serves as an attending, prior to fellowship. We chose to focus on the fellow perspective, hypothesizing that existing support systems, such as orientations, already reflect the gaps that fellowship program directors perceive incoming fellows to have, and that fellows, who themselves are experiencing the transition, could provide firsthand perspective on the salient features of their transition.

## Conclusion

We found that even advanced trainees face challenges on transition to advanced training programs. By exploring these specific challenges, we illuminate ways to better support the transition.

## Additional File

The additional file for this article can be found as follows:

10.5334/pme.1654.s1 Supplementary File 1. Interview Guide.
