# Supplementary material for: Novice Experts: Exploring Fellows’ Perspectives on the Transition from Residency to Fellowship
Source: Perspect Med Educ. 2025 Feb 14;14(1):66–73. doi: 10.5334/pme.1654 (PMC11827558; doi:10.5334/pme.1654)
Supplement: Supplementary File 1. — Interview Guide. [file pme-14-1-1654-s1.pdf]

Supplementary File– Interview Guide

1. Where did you train for residency?
2. Tell me about your experience of transitioning from resident to fellow.
    a. *Probe: tell me about an experience that [most] went really well that reflects this transition or tell me about a particularly challenging experience that reflects this transition*
    b. Probe – What made it easier?
    c. Probe- What made it harder?
3. What is the role of a fellow? Are there differences with residency? Why?
4. In developing your skillset as a *** fellow, what has been helpful or supportive? Why and how? What were Barriers? Why and how?
    a. *Probe: Probe: tell me about an experience that went really well that reflects your development as a *** or tell me about a particularly challenging experience that reflects this development as a ***?*

Optional probes: contribution of any of the following based on the study describing contextual competence at the residency rotation transition:

    b. Physiological needs/ practical needs -What supported your navigation of practical needs? Inhibited?
    c. Legitimacy/belonging – What factors supported your feeling a part of the team? Inhibited?
    d. Scope of role – How did the scope of the role change from residency to fellowship?
    e. Responsibilities – How did your responsibilities evolve? Were you prepared for these new responsibilities?
    f. How to get things done – What supported your being able to get things done? Inhibited?

5. How did supervisors know that they could entrust you in your new role?
6. How did supervisors support you in the new role? Impede?
7. Which feedback/assessment was particularly helpful in your development? Harmful?
8. Is there anything else you’d like to share about how you developed competence in a new context?
